# Influence of Sodium Lauryl Sulfate and Tween 80 on Carbamazepine–Nicotinamide Cocrystal Solubility and Dissolution Behaviour

**DOI:** 10.3390/pharmaceutics5040508

**Published:** 2013-10-11

**Authors:** Mingzhong Li, Ning Qiao, Ke Wang

**Affiliations:** School of Pharmacy, De Montfort University, Leicester LE1 9BH, UK; E-Mails: p10510892@myemail.dmu.ac.uk (N.Q.); kwang@dmu.ac.uk (K.W.)

**Keywords:** surfactant, carbamazepine–nicotinamide cocrystal, UV imaging, intrinsic dissolution rate, Raman spectroscopy

## Abstract

The influence of the surfactants of sodium lauryl sulfate (SLS) and Tween 80 on carbamazepine–nicotinamide (CBZ–NIC) cocrystal solubility and dissolution behaviour has been studied in this work. The solubility of the CBZ–NIC cocrystal was determined by measuring the eutectic concentrations of the drug and the coformer. Evolution of the intrinsic dissolution rate (IDR) of the CBZ–NIC cocrystal was monitored by the UV imaging dissolution system during dissolution. Experimental results indicated that SLS and Tween 80 had little influence upon the solubility of the CBZ–NIC cocrystal but they had totally opposite effects on the IDR of the CBZ–NIC cocrystal during dissolution. SLS significantly increased the IDR of the CBZ–NIC cocrystal while Tween 80 decreased its IDR.

## 1. Introduction

Pharmaceutical cocrystals as a new class of solid drugs with improved physicochemical properties have attracted increased interest from both industry and academic researchers [1,2,3]. However, like many other solubility enhancement methods (such as salt formation, forming an amorphous solid or dispersion, or using a metastable polymorph) cocrystal formation can result in a phenomenon called solution-mediated phase transformation (SMPT) which is crystallisation of a stable solid phase during dissolution of a metastable phase caused by supersaturated conditions either in solution or at the surface of the dissolving solid [4,5,6,7]. Recently, research in our laboratory has investigated the dissolution and transformation behaviour of the carbamazepine–nicotinamide (CBZ–NIC) cocrystal using *in situ* techniques of UV imaging and Raman spectroscopy, indicating that the conversion of the CBZ–NIC cocrystal to its dihydrate form during dissolution can significantly reduce the advantages of the improved drug solubility and dissolution rate [8].

Several approaches have been investigated to inhibit the SMPT of active pharmaceutical ingredients (APIs), such as the inclusion of impurities in the formulation [9,10,11] and the solubilisation of poorly soluble drugs by surfactants to increase solubility and dissolution rates [12,13,14,15,16,17]. A recent study by Rodriguez-Hornedo and co-workers has demonstrated that surfactants can impart thermodynamic stability to cocrystals that otherwise convert to the parent drug solid in aqueous solutions [18,19,20].

Intrinsic dissolution rate (IDR) is an important property of a drug compound, which measures the rate of dissolution of a pure drug substance from a constant surface area. Therefore, IDR is independent of formulation effects and measures the intrinsic properties of the drug as a function of dissolution media, e.g., pH, ionic strength and counter-ions. Recently, UV imaging has shown great promise in understanding the dissolution behaviour of drug compounds because, when combined with a channel flow cell method, it can visualise the solution concentration distribution during dissolution in real time, providing a platform for generating spatial and temporal information about the dissolved solution phase of a solid drug during dissolution [8,21,22].

The role of a surfactant on the IDR of a cocrystal has been virtually unexplored. In order to critically evaluate and understand the role of surfactants in inhibiting the SMPT of a cocrystal, two different surfactants (sodium lauryl sulfate and Tween 80) were selected to investigate their effects on the solubility and intrinsic dissolution rate of the carbamazepine–nicotinamide (CBZ–NIC) cocrystal. Sodium lauryl sulfate (SLS, also known as Sodium dodecyl sulfate, SDS) is an anionic surfactant and Tween 80 (Polysorbate 80) is a nonionic surfactant, both of which have been widely used in various drug dosage forms to control wetting, stability, and solubilisation of hydrophobic drugs. Carbamazepine (CBZ) is a poorly water-soluble compound with a narrow range of therapeutic efficacy. CBZ exists as two principal polymorphs (forms III and I) which constitute an enantiotropic pair. CBZ form III (CBZ III) is thermodynamically the most stable form at room temperature and is used in the formulation of marketed products. CBZ dihydrate (CBZ DH) is the most stable form in aqueous solutions, and anhydrous CBZ forms undergo a transformation to CBZ DH upon contact with water. This contributes to the bioequivalence and clinical failures of some CBZ generic tablets [23]. Nicotinamide (NIC) is one of the popular coformers used to study pharmaceutical cocrystals and can form cocrystals with many pharmaceutical active ingredients [24]. Determination of cocrystal solubility is not straightforward. The true solubility of a cocrystal may be difficult to measure because of the transformation of the more stable pure drug form when exposed to solvent (as described above). The apparent solubility of the CBZ–NIC cocrystal at different concentrations of surfactants was predicted from measurement of eutectic points or transition concentrations where the solid cocrystal and the drug are in equilibrium with the solution [25,26,27]. The evolution of the intrinsic dissolution rate of the CBZ–NIC cocrystal at different concentrations of surfactants was monitored by the UV imaging dissolution system during dissolution. The solid-state changes of CBZ–NIC cocrystal after dissolution were quantified by Raman spectroscopy. Comparison of the solubility and intrinsic dissolution behaviour of the CBZ polymorphs (form III and dihydrate) and the physical mixture of CBZ form III and NIC were also given.

## 2. Experimental Section

### 2.1. Materials

Anhydrous carbamazepine (CBZ III), nicotinamide (NIC) (≥99.5% purity) were obtained from Sigma–Aldrich (Sigma–Aldrich Company Ltd., Dorset, UK) and used as received. Tween 80, sodium lauryl sulfate (SLS) (>99%) and methanol (HPLC grade) were obtained from Fisher Scientific (Fisher Scientific, Loughborough, UK) and used as received. Double-distilled water was generated from a Bi-Distiller (WSC044.MH3.7, Fistreem International Limited, Loughborough, UK) and used throughout the study. Carbamazepine dihydrate (CBZ DH), 1:1 carbamazepine–nicotinamide (CBZ–NIC) cocrystal and equimolar physical mixture of CBZ III and NIC were prepared as previously reported [8].

### 2.2. Methods

#### 2.2.1. Apparent Solubility of CBZ in Water and Surfactant Solutions

The apparent solubility of CBZ of CBZ DH, the CBZ–NIC cocrystal, and the physical mixture in both water and surfactant solutions were determined using an air-shaking bath method. Excess of CBZ DH, cocrystal and physical mixture were added into a small vial containing 30 mL of media and vortexed for 20 s. The vials were placed in a horizontal air-shaking bath at 25 °C at 100 rpm for 72 h. Aliquots were filtered through 0.45 µm filters (Thermo Scientific Nalgene, Rochester, NY, USA) and diluted properly for determination of the concentrations of CBZ and NIC by HPLC. A series of surfactant solutions were prepared: 0.35, 1.7, 3.5, 6.9, 10.4, 17.3, 34.7 mM SLS solutions; and 0.076, 0.76, 1.5, 2.3, 3.8, 7.6, 17.3, 34.7 mM Tween 80 solutions. Solid residues were retrieved from the solubility tests and air-dried for 30 min. All of dried solids were characterized by FTIR, Raman and DSC. All solubility tests were carried out in triplicate.

#### 2.2.2. Cocrystal Eutectic Points

Cocrystal eutectic points or transition concentrations were measured as a function of SLS or Tween 80 concentration in water at 25 ± 0.5 °C [25,26]. In this work, the eutectic concentrations of CBZ and NIC were determined by adding excess CBZ DH in 5 mL of a solution of mixture of surfactant and near saturated NIC for 72 h. Solid phases at equilibrium were confirmed by FTIR-ATR, Raman and DSC. All tests were carried out in triplicate. For 1:1 AB cocrystal without consideration of ionization for either component, its aqueous solubility was determined from eutectic concentration measurements by [25],

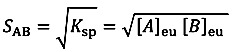
(1)
where *K*_sp_ is the cocrystal solubility product and [*A*]_eu_ and [*B*]_eu_ are the eutectic concentrations of drug and coformer at equilibrium.

#### 2.2.3. Critical Micelle Concentration and Solubilisation Capacity

The critical micellar concentrations (CMCs) of SLS and Tween 80 were determined in aqueous solutions in the presence of saturated carbamazepine from the surface tension dependence on surfactant concentration a KSV CAM200 surface tension meter at room temperature. The instrument was calibrated by a calibration ball with 6 mm in diameter for water. The CMC of a surfactant was determined from the break in the curve of surface tension *versus* log of surfactant concentration. All tests were carried out in triplicate.

The molar solubilisation capacities of SLS and Tween 80 for CBZ can be obtained according to the following equation:

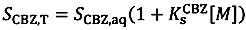
(2)
where *S*_CBZ,T_ is the solubility of CBZ in micellar solution; *S*_CBZ,aq_ is the solubility of CBZ in water; [*M*] = *C* − *CMC* is the surfactant micellar concentration; and *C* is the surfactant concentration.

#### 2.2.4. Intrinsic Dissolution Study by UV Imaging System

**Figure 1:**
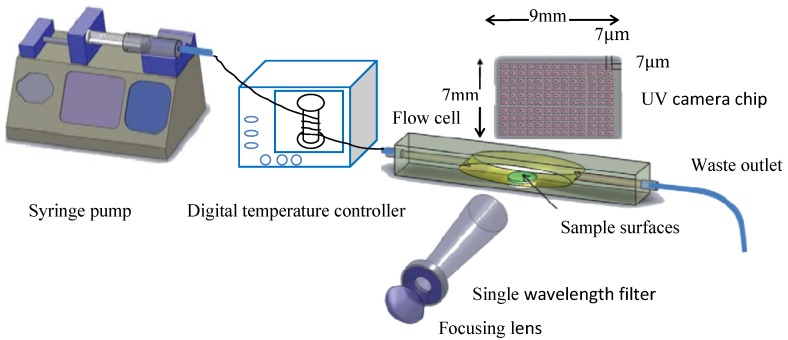
Schematic UV imaging dissolution system.

The dissolution behaviour of CBZ III, the CBZ–NIC cocrystal, and the physical mixture of CBZ III and NIC in both pure water and at different concentrations of surfactant solutions was studied by an ActiPix SDI 300 UV surface imaging system (Paraytec Ltd., York, UK). It comprises of a sample flow cell, syringe pump, temperature control unit, UV lamp and detector, and control and data analysis system, shown in Figure 1. Details of a description of the system and selection of the filter can be found in our previous study [8]. UV imaging calibration was performed by imaging a series of CBZ standard solutions in pure water with concentrations of 4.23 × 10^−3^ mM, 2.12 × 10^−2^ mM, 4.23 × 10^−2^ mM, 8.46 × 10^−2^ mM, 1.69 × 10^−1^ mM, and 2.54 × 10^−1^ mM. The standard curve was constructed by plotting the absorbance against concentration of each standard solution. The calibration curve was validated by a series of CBZ standard solutions in different surfactant concentration solutions, showing that SLS and Tween 80 did not affect the accuracy of the model and the calibration curve was applicable for the dissolution test with surfactant solutions. Experiments were repeated three times for construction of a UV calibration curve. The sample compact in a dissolution test was made by filling around 5 mg of the sample into a stainless steel cylinder (inner diameter: 2 mm) and compressed by a Quickset MINOR torque screwdriver (Torqueleader, M.H.H. engineering Co. Ltd., Guildford, UK) for 1 min at a constant pressure of 40 cN.m. All dissolution tests were performed at 25 ± 0.5 °C in triplicate. The concentrations of each surfactant used in the study were: 0.35 and 10.4 mM of SLS solutions and 0.076 and 7.6 mM of Tween 80 solutions.

#### 2.2.5. High Performance Liquid Chromatography (HPLC)

The concentrations of CBZ and NIC in a solution were analysed by Perkin Elmer series 200 HPLC system. A HAISLL 100 C18 column (5 µm, 250 × 4.6 mm) (Higgins Analytical Inc., Mountain View, CA, USA) at ambient temperature was used. The mobile phase composed of 70% methanol and 30% water, and the flow rate was 1 mL/min using an isocratic method.

#### 2.2.6. Light Microscopy

The sample compacts were analysed prior to and after a UV imaging dissolution test using a LEICA DM 750 microscope equipped with a lens of ten-time magnification, infinity 2-1C camera and Studio Capture Video microscopy software (version 4; Studio86Designs, Lutterworth, UK).

#### 2.2.7. Raman Spectroscopy

The sample compacts were analysed prior to and immediately after UV imaging dissolution tests using an EnSpectr R532^®^ Raman spectrometer (Enhanced Spectrometry Inc., Torrance, CA, USA). The integration time was 200 milliseconds and each spectrum was obtained based on an average of 50 scans. In order to quantify the percentage of CBZ DH crystallised during dissolution, CBZ III and CBZ–NIC cocrystal were blended separately with CBZ DH to form binary physical mixtures at 20% (*w*/*w*) intervals from 0% to 100% of CBZ DH in the test samples. Each sample was prepared in triplicate and measured by Raman spectroscopy. Ratios of the characteristic spectral intensities (based on our previous study [8]) were used to construct the calibration models.

#### 2.2.8. FTIR-ATR Spectrometer

IR spectra of CBZ DH, NIC, CBZ III, the CBZ–NIC cocrystal and the physical mixture of CBZ III and NIC were obtained by an ALPHA A4 sized Benchtop FTIR-ATR spectrometer (Bruker UK Limited, Coventry, UK). Measurement settings are: resolution 2 cm^−1^, data range 4000–400 cm^−1^.

## 3. Results and Discussion

### 3.1. Results of Solubilisation by Surfactants

**Figure 2:**
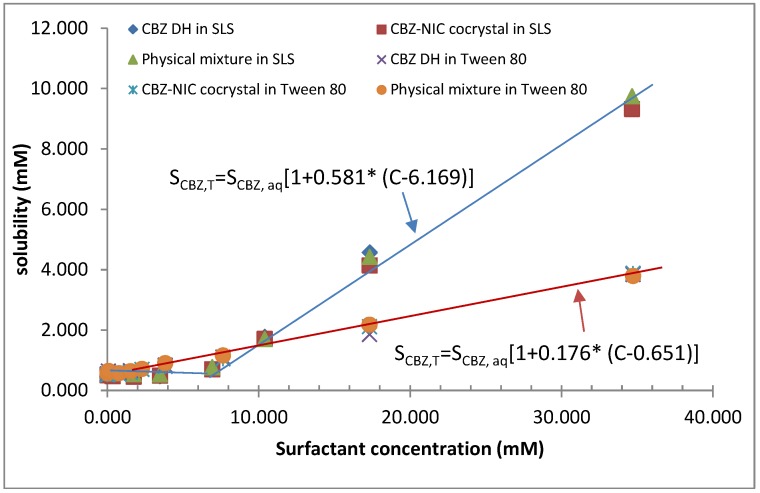
Apparent solubility of CBZ as a function of surfactant concentration.

The apparent solubility of CBZ of CBZ DH (CBZ III), the CBZ–NIC cocrystal and the physical mixture of CBZ III and NIC in water, and at different concentrations of SLS and Tween 80 after 72 h are shown in Figure 2 and details of data are in Table S1 in Supplementary material. Generally, there was no significant difference in the apparent solubility of CBZ between the parent drug, CBZ, the cocrystal and the physical mixture. FTIR, Raman and DSC data (not shown) of the solid residue indicated that the CBZ–NIC cocrystal had totally converted to CBZ DH; therefore, the same apparent solubility of CBZ of the cocrystal and CBZ DH was found.

Based on the measured solubility data of CBZ, the molar solubilisation capacities and CMCs of SLS and Tween 80 can be estimated, as summarised in Table 1. It is shown that the estimated CMCs of SLS and Tween 80 are in good agreement with those measured by the surface tension technique. The change of surface tension at different surfactant concentrations of aqueous solutions in the presence of saturated CBZ is shown in Figure S1 in Supplementary material. Tween 80 has a higher solubilisation capacity at a low concentration due to its lower CMC. However, the anionic surfactant SLS showed a dramatic solubilizing ability with CBZ when its concentration was higher than its CMC. When the concentration of SLS is higher than a critical value of 8.567 mM, SLS has a higher solubilisation capacity for CBZ than Tween 80, which can be seen from the solubility data of CBZ in Figure 2.

**Table 1:** Estimated molar solubilisation capacity and CMC.

Surfactant	Solubilisation capacity (mM^−1^)	CMC estimated (mM)	CMC measured (mM)
SLS	0.581	6.196	5.5
Tween 80	0.176	0.651	0.012–1.05

**Figure 3:**
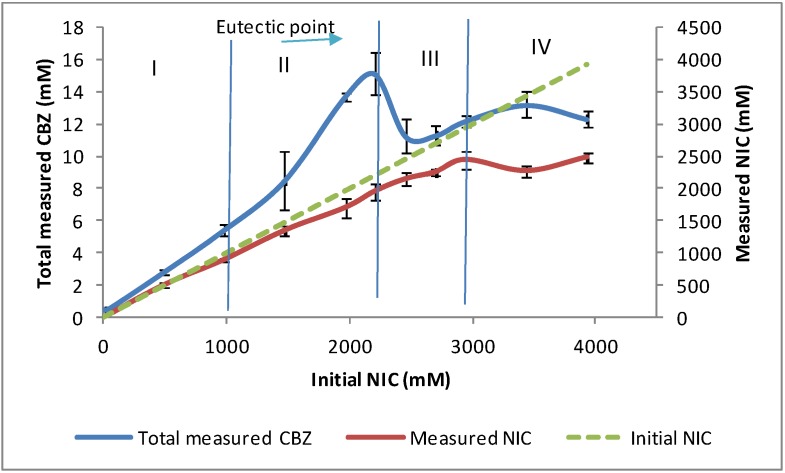
Apparent CBZ solubility profile as a function of NIC concentration after 72 h.

The apparent solubility of CBZ was measured as a function of the coformer NIC concentration in order to determine CBZ–NIC cocrystal eutectic point (shown in Figure 3), in which four regions can be identified:

*Region I*: from 0 to 1000 mM initial NIC concentrations, the apparent solubility of CBZ increases rapidly with increasing concentration of NIC, indicating the 1:1 complex formation of CBZ and NIC. The apparent solubility of CBZ can increase up to 10 times in comparison to apparent CBZ solubility in water. In this region, the solubility limit of the complex formed is not exceeded and the solid residue should be CBZ DH only, which was confirmed by DSC, FTIR and Raman analyses (Figures S2–S4 in the Supplementary material). In this region, the measured concentration of NIC was the same as that of the initial NIC.

*Region II*: from 1000 to 2400 mM initial NIC concentrations, the apparent solubility of CBZ increases at a higher rate. When the apparent solubility limit of CBZ of the 1:1 complex formed was exceeded in this region, the CBZ–NIC cocrystal precipitated. In the meantime, higher order complexes of CBZ and NIC were generated, resulting in a further 18-fold increase in the apparent CBZ solubility in water. The solid residue indicated the presence of two phases of CBZ–NIC cocrystal and CBZ DH confirmed by DSC, FTIR and Raman analyses (Figures S2–S4 in the Supplementary material). According to the definition, any point in the region II should be a eutectic point of CBZ–NIC cocrystal. In this study, we set the highest total CBZ concentration point as its eutectic point shown in Figure 3 as [*CBZ*]_eu_ = 15.1 mM and [*NIC*]_eu_ = 1956.8 mM.

*Region III*: from 2400 to 3000 mM initial NIC concentrations, the concentration of CBZ decreases with increasing NIC concentration in solution. In this region, all of the solid CBZ DH was consumed to form the complex to generate the supersaturated solution of the complex, resulting in precipitation of large amount of CBZ–NIC cocrystal and reduction of CBZ concentration in solution. For this reason, the overall CBZ concentration decreased in the solution shown in Figure 3. It has been shown that the CBZ–NIC cocrystal and NIC are coexisting in the solid residues, shown in DSC, FTIR and Raman analyses (Figures S2–S4 in the Supplementary material).

*Region IV*: Above 3000 mM initial NIC concentration, the concentrations of CBZ and NIC in the solution are constant. The solid residues indicated the presence of two phases of CBZ–NIC cocrystal and NIC, confirmed by DSC, FTIR and Raman analyses (Figures S2–S4 in the Supplementary material).

Table 2 shows the total concentrations of CBZ and NIC at the eutectic points of different concentrations of SLS and Tween 80. Compared with the eutectic point in water, the apparent solubility of CBZ increased slightly with an increase in SLS concentration while the concentration of INC was constant with SLS concentration (which was slightly smaller than that in pure water). According to the solubility definition of a 1:1 cocyrstal in Equation 1, the solubility of the CBZ–NIC cocrystal at different concentrations of SLS is shown in Table 2. The solubility of the CBZ–NIC cocrystal was nearly constant and same as that in water when the SLS concentration was below its CMC and it increased slightly when the SLS concentration was above its CMC.

**Table 2:** CBZ–NIC cocrystal eutectic point, cocrystal solubility and solubility ratio.

Solvent concentration (mM)		[*CBZ*]_eu_ (mM)	[*NIC*]_eu_ (mM)	Cocrystal solubility *S*_cc_ (mM)	Solubility ratio *S*_cc_/*S*_CBZ,aq_
Water		15.10 ± 1.32	1,956.8 ± 126.8	171.9	319
SLS	0.35	15.9 ± 1.8	1,665.2 ± 62.6	162.8	302
	1.7	16.3 ± 0.76	1,807.9 ± 90.3	171.5	319
	3.5	17.6 ± 0.63	1,818.9 ± 57.0	178.7	332
	6.7	17.5 ± 0.65	1,914.0 ± 136.3	183.2	340
	10.4	17.7 ± 0.42	1,811.1 ± 65.7	179.2	333
	17.3	18.1 ± 0.70	1,934.6 ± 51.8	187.3	348
	34.7	16.1 ± 2.77	1,839.5 ± 255.9	171.8	319
Tween 80	0.076	15.5 ± 0.68	1,847.3 ± 15.6	169.5	315
	0.76	15.9 ± 0.37	1,852.3 ± 56.9	171.7	319
	1.5	16.0 ± 0.70	2,024.3 ± 50.9	180.2	335
	2.3	17.4 ± 1.65	1,853.3 ± 109.9	179.5	334
	3.8	16.75 ± 2.78	1,624.5 ± 69.1	164.9	306
	7.6	14.98 ± 0.45	1,691.1 ± 87.4	159.2	296
	17.3	15.33 ± 0.89	1,638.9 ± 79.8	158.5	294

Compared with the eutectic point in water, the apparent solubility of CBZ was almost constant with an increase in Tween 80 concentration, while the concentration of NIC decreased at higher concentration of Tween 80 solutions. The solubility of the CBZ–NIC cocrystal was nearly same as that in water at a lower concentration of Tween 80 solution and then decreased slightly when the Tween 80 concentration increased (see Table 2).

### 3.2. Results of Intrinsic Dissolution Rate

**Figure 4:**
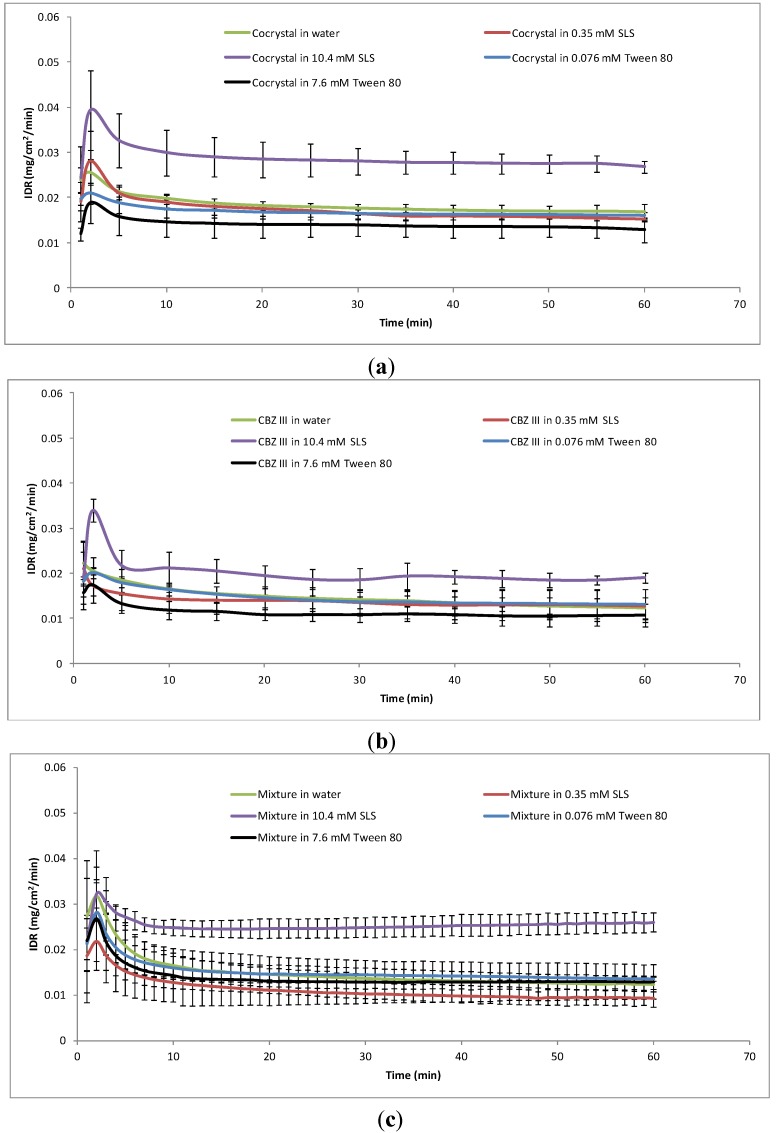
Dissolution profiles of test samples at different dissolution media. (**a**) CBZ–NIC cocrystal; (**b**) CBZ III; (**c**) equimolar physical mixture of CBZ III and NIC.

The IDR profiles from the compacts of the pure CBZ III, the CBZ–NIC cocrystal, and the physical mixtures of CBZ III and NIC at different dissolution media are shown in Figure 4. A video clip of CBZ–NIC cocrystal dissolution at 10.4 mM SLS solution can be found in the Supplementary material. All IDR profiles show the same trend: (1) within the first 2 min the IDR of a test sample reached its maximum value and then decreased quickly within 10 min; (2) after 20 min of dissolution, all IDRs of the test samples reached static values. The IDR of each sample during dissolution in each dissolution medium was decreasing, indicated by the growth of solid material on the surface of the sample compact that can be visualised by light microscopy (see Table 3). Prior to the dissolution tests, all of the compact surfaces were smooth. After the dissolution tests, the microscope images show that small needle-shaped crystals have appeared on the compact surfaces, indicating that the solid-state changes due to crystallisation of CBZ DH from the supersaturated solutions on the compact surfaces.

The IDRs of the CBZ–NIC cocrystal are shown in Figure 4a. It was found that the IDR profile of CBZ in the 0.35 mM SLS dissolution medium was almost identical to that in water. In the 0.076 mM Tween 80 dissolution medium, its dissolution profile was slightly lower than in pure water. In the 10.4 mM SLS dissolution medium, the IDR profile of the CBZ–NIC cocrystal increased significantly in comparison with that in water, indicating that SLS can increase the cocrystal dissolution rate when its concentration was higher than its CMC. To our surprise, in the 7.6 mM Tween 80 dissolution medium, the CBZ dissolution profile was much lower than that in water.

**Figure 5:**
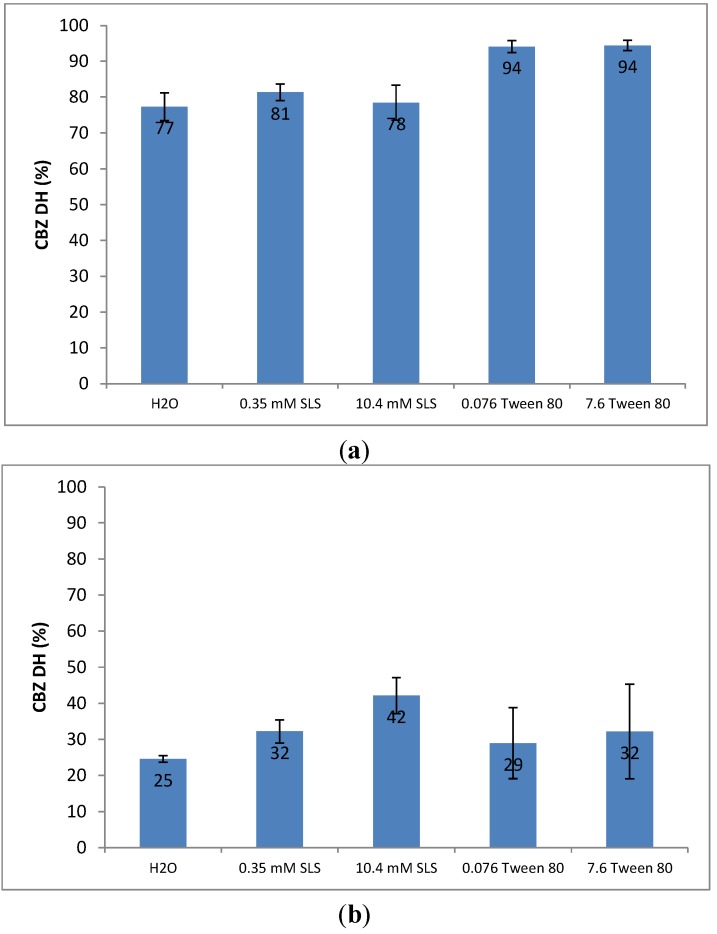
Comparison of percentages of CBZ DH on the surfaces of sample compacts after dissolution test. (**a**) CBZ–NIC cocrystal; (**b**) CBZ III.

Raman spectroscopy was used before and after UV imaging to characterise the solid state (see Figure S5 in the Supplementary materials), and showed some significant changes before and after the dissolution tests, which are in good agreement with previous findings [8]. In order to quantify the solid-state change during dissolution, a calibration model was built to quantify the percentage of CBZ DH on the surface of a sample compact after dissolution [8]. The percentage of CBZ DH covering on the surface of sample compact for each dissolution test is shown in Figure 5a. Interestingly the percentages of CBZ DH crystals on the surfaces of the CBZ–NIC cocrystal compacts in the 0.35 mM SLS and 10.4 mM SLS dissolution media are nearly constant (*i.e*., 81% in 0.35 mM SLS solution and 78% in 10.4 mM SLS dissolution solution), which nearly equal to that (*i.e*., 77%) of the CBZ–NIC cocrystal in water. By contrast, the percentages of CBZ DH covering the surfaces of the CBZ–NIC cocrystal compacts in both 0.076 mM Tween 80 and 7.6 mM Tween 80 (*i.e*., 94% in both Tween 80 solutions) are much higher than that of CBZ–NIC cocrystal in water.

CBZ III is an anhydrous form of CBZ and has similar dissolution behaviour to that of the CBZ–NIC cocrystal in different surfactant solutions. If a surfactant concentration was less than its CMC, the IDR profile of CBZ III in the 0.35 mM SLS dissolution or 0.076 mM Tween 80 was similar to that in water, as shown in Figure 4b. When compared with the dissolution behaviour of CBZ III in water, the IDR profile of the CBZ III increased significantly in the 10.4 mM SLS dissolution medium and decreased in the 7.6 mM Tween 80 dissolution medium. The percentage of CBZ DH covering on the surface of sample compact for each dissolution test based on Raman spectra is shown in Figure 5b. The percentages of CBZ DH crystals on the surfaces of CBZ III compacts increased significantly with SLS concentration in comparison with that in pure water. A similar trend was found in a Tween 80 dissolution medium: the percentages of CBZ DH covering the surfaces of CBZ III compacts in both 0.076 mM Tween 80 and 7.6 mM Tween 80 dissolution media increased, but to a lesser extent than those in the SLS dissolution media.

Evolution of the IDRs of the equal molar physical mixture of CBZ III and NIC at different dissolution media is shown in Figure 4c. The biggest variability of the IDRs of the physical mixture is shown, which is consistent with that of dissolving polyphase mixtures in which the more soluble component dissolves more rapidly from the surface of the compact, leaving behind a porous layer of the less soluble component [28,29]. Light microscopy showed that holes appeared on the compact surface due to dissolved NIC (see Table 3), resulting in a significant change of the dissolution rate. Similar trends of effects of the dissolution media on the IDR of physical mixture was found with the 10.4 mM SLS dissolution medium, the IDR profile increased significantly compared with that of pure water in Figure 4c.

**Table 3:** Light microscopy photographs of the sample compacts before and after dissolution tests in 10.4 mM SLS and 7.7 mM Tween 80 dissolution media.

Sample	Surfactant	Before	After
CBZ–NIC cocrystal	10.4 mM SLS	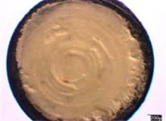	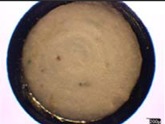
	7.6 mM Tween 80	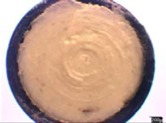	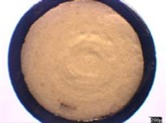
CBZ III	10.4 mM SLS	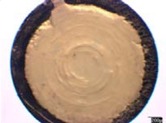	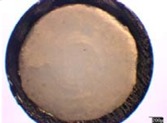
	7.6 mM Tween 80	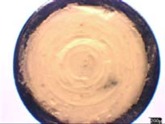	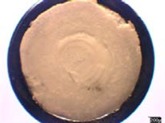
Physical mixture of CBZ III and NIC	10.4 mM SLS	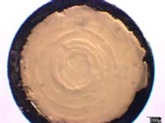	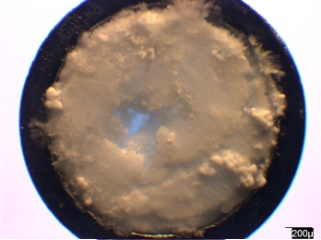
	7.6 mM Tween 80	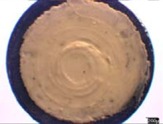	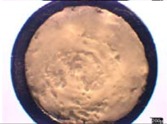

### 3.3. Discussion of Solubility Enhancement by Surfactants

From the study, it has been shown that the CBZ–NIC cocyrstal has the same apparent solubility of CBZ as that of CBZ DH (CBZ III) because of the dissociation of the individual components of CBZ–NIC cocrystal, which in turn is due to the weak hydrogen bonds in the molecular assembly of the two components of CBZ and NIC. There was no significant effect of the cocrystal coformer NIC on the apparent solubility of CBZ because of a low concentration of NIC in the solution. Based on the apparent CBZ solubility profile as a function of NIC concentration, it has been shown in Figure 3 that at a low NIC concentration (up to 40 mM) the apparent solubility of CBZ was constant.

The phase solubility diagram of CBZ in the presence of NIC has shown that the apparent CBZ solubility has been enhanced significantly and in a nonlinear fashion as a function of NIC concentration (Figure 3). The experimental observations can be explained by the stacking complexation mechanism of NIC with CBZ [30], in which linear increases in the apparent solubility of CBZ may be attributed to the formation of 1:1 complexes in *Region I* and nonlinear increases in *Region II*, indicating that higher order complexes were formed due to self-association of NIC. Due to the strong interaction of CBZ and NIC in the solution, the eutectic point of the CBZ–NIC cocrystal was not a single point, but covered the whole range of *Region II* from 1000 mM to 2400 mM of NIC concentrations. In this study, the highest total measured CBZ concentration was selected as the eutectic point of the CBZ–NIC cocrystal (Figure 3), showing that the solubility ratio of *S*_cc_/*S*_CBZ,aq_ was 319. If the lowest total measured CBZ concentration in the *Region II* was selected as the eutectic point of the CBZ–NIC cocrystal, the solubility ratio of *S*_cc_/*S*_CBZ,aq_ was 130. The reported value of the solubility ratio of *S*_cc_/*S*_CBZ,aq_ was 152, which was in the *Region II* of the phase solubility diagrams [25].

SLS and Tween 80 have little effects on enhancing the solubility of the CBZ–NIC cocrystal in Table 2, in which the solubility of the CBZ–NIC cocrystal increased slightly with SLS concentration and decreased with Tween 80 concentration; however, significant solubility enhancement for CBZ DH by SLS was observed above its CMC.

### 3.4. Discussion of Effects of Surfactants on SMPT of CBZ III and CBZ–NIC Cocrystal

Comparing the IDRs of CBZ III, the CBZ–NIC cocrystal and the physical mixture of CBZ III and NIC in the same dissolution medium, it has been shown that CBZ–NIC cocrystal has the highest IDR in Figure 4, indicting the advantages of the CBZ–NIC cocrystal in enhancing the dissolution rate [8].

From this study, it has also shown that the SMPT of CBZ III and the CBZ–NIC cocrystal can be altered by the inclusion of a surfactant in the dissolution medium. However, CBZ III and the CBZ–NIC cocrystal have shown different transformation behaviour affected by different surfactants.

It is well known that surfactants can enhance dissolution of poorly water-soluble drugs in two ways, *i.e.*, either by lowering the surface tension at the solid drug surface to increase the surface area available for dissolution or by increasing drug solubility [31]. The ability of surfactants of SLS and Tween 80 to manipulate the SMPT of CBZ–NIC cocrystals and CBZ III affects the nucleation and/or growth of the new crystalline phase of CBZ DH.

Dissolution experiments with CBZ III have shown the increased percentages of CBZ DH precipitated on the surfaces of the sample compacts with increasing surfactant concentrations of SLS and Tween 80 (see Figure 5b), indicating that both SLS and Tween 80 facilitated the surface-mediated nucleation of CBZ DH on the dissolving CBZ III. These results are in line with classic nucleation theory which predicts that crystal nucleation increases with decreasing interfacial tension by the inclusion of surfactants in the dissolution medium [32]. Comparing the two surfactants, the anionic surfactant SLS has shown a more significant effect on nucleation of CBZ DH during dissolution of CBZ III than Tween 80, indicated by the higher percentages of CBZ DH covering on the surface of the dissolution compact.

Dissolution experiments of the CBZ–NIC cocrystal with and without SLS show the same percentage of CBZ DH precipitated on the surface of each sample compact (Figure 5a), indicating that SLS does not facilitate the surface-mediated nucleation of CBZ DH on the dissolving CBZ–NIC cocrystal. The rate of CBZ DH nucleation for the CBZ–NIC cocrystal dissolution was influenced mainly by the coformer NIC, rather than as a consequence of decreasing the interfacial tension by SLS. Dissolution experiments of the CBZ–NIC cocrystal with 0.076 and 7.6 mM concentrations of Tween 80 have shown that significant higher of CBZ DH was precipitated as a covering on the sample compact surfaces, (94% compared with 77% of CBZ DH crystallised on the surface in pure water), indicating that the rate of CBZ DH nucleation during CBZ–NIC cocrystal dissolution was affected by both the coformer NIC and by Tween 80.

Dissolution rate is the net result of solubilisation rates of the original solid phase and the formation rates of a less soluble solid phase. SLS has a flexible alkyl chain that exhibits axial polarity (hydrophilic head group and hydrophobic tail) and a well-defined CMC. The transformation process of the CBZ–NIC cocrystal and CBZ III to CBZ DH is sensitive to the SLS concentration in a dissolution medium. The solubility of CBZ for solid of CBZ III has not increased in 0.35 mM SLS dissolution medium below its CMC. Due to increasing in rate of CBZ DH nucleation, the IDR profile of CBZ III in the 0.35 mM SLS dissolution medium was lower than its corresponding profile in water (Figure 4b). Because SLS does not influence CBZ DH nucleation for the dissolving CBZ–NIC cocrystal, the IDR profile of the CBZ–NIC cocrystal in the 0.35 mM SLS dissolution medium was almost same as its corresponding profile in water (Figure 4a). The solubility of CBZ for solids of CBZ III and the CBZ–NIC cocrystal have increased significantly when the concentration of SLS is higher than its CMC due to the inclusion of CBZ molecules into the surfactant micelles, resulting in a lack of growth material of CBZ in the solution which retards crystallization and inhibits growth of the stable crystal form CBZ DH on the compact surface. The IDR of CBZ III in the 10.4 mM SLS solution was increased 1.3 times relative to that in pure water whilst the IDR of the CBZ–NIC cocrystal in the same solution was increased to 1.6 times of its corresponding value in pure water.

Tween 80 is a non-ionic surfactant and has a wide range of CMC values because of progressive association and heterogeneous distribution of aggregates. Although Tween 80 can increase the solubility of CBZ in solution, its solubilisation capacity was limited—the large molecule sizes of Tween 80 and its aggregates in the solution mean it can form an interfacial barrier to prevent CBZ molecules getting into the bulk solution, resulting in accelerated nucleation and growth of the stable crystal form CBZ DH on the sample compact surface. Therefore, the experimental results in this work indicated that Tween 80 had the opposite effect on the IDRs of CBZ III and the CBZ–NIC cocrystal as those of SLS, *i.e.*, the inclusion of Tween 80 in the dissolution medium reduced the IDRs of CBZ III and CBZ–NIC cocrystal (Figure 4).

## 4. Conclusions

The influence of two surfactants (SLS and Tween 80) on the solubility and dissolution behaviour of the CBZ–NIC cocrystal, CBZ III and the physical mixture of CBZ III and NIC has been investigated in this study. From the study, it has been shown that the CBZ DH (CBZ III), the CBZ–NIC cocrystal and the physical mixture of CBZ III and NIC have the same apparent CBZ solubility. SLS and Tween 80 have little effect on the solubility of the CBZ–NIC cocrystal in comparison with the significant solubility enhancement for CBZ DH by SLS above its CMC.

From this study, it has shown that the SMPT of CBZ III and the CBZ–NIC cocrystal can be altered by the inclusion of a surfactant in the dissolution medium. However, CBZ III and the CBZ–NIC cocrystal have shown different transformation behaviour with different surfactants. The IDR profile of CBZ III and the CBZ–NIC cocrystal increased significantly when the concentration of SLS was higher than its CMC in a dissolution medium. The inclusion of Tween 80 in the dissolution medium reduced the IDRs of CBZ III and the CBZ–NIC cocrystal due to its large molecule size which can form an interfacial barrier to prevent the CBZ molecules getting into the bulk solution, resulting in accelerated nucleation and growth of the stable crystal form CBZ DH on the sample compact surface.

## Supplementary Files

Supplementary File 1 Supplementary Information (PDF, 281 KB)
