# Supplementary material for: Influence of Sodium Lauryl Sulfate and Tween 80 on Carbamazepine–Nicotinamide Cocrystal Solubility and Dissolution Behaviour
Source: Pharmaceutics. 2013 Oct 11;5(4):508–24. doi: 10.3390/pharmaceutics5040508 (PMC3873677; doi:10.3390/pharmaceutics5040508)
Supplement: Supplementary File 1 — Supplementary Information (PDF, 281 KB) [file pharmaceutics-05-00508-s001.pdf]

Supplementary Information

Table S1. Equilibrium solubility of CBZ DH, Cocrystal, and physical mixture at 25 °C as a function of surfactant concentration after 72 h.

	Concentration	CBZ DH	Cocrystal		Physical mixture	
	mM	CBZ (mM)	CBZ (mM)	NIC (mM)	CBZ (mM)	NIC (mM)
SLS	0.000 (water)	0.538 ± 0.038	0.502 ± 0.028	2.613 ± 0.140	0.575 ± 0.036	2.569 ± 0.164
	0.347	0.476 ± 0.020	0.473 ± 0.030	2.482 ± 0.162	0.550 ± 0.024	2.776 ± 0.042
	1.734	0.457 ± 0.015	0.450 ± 0.041	2.382 ± 0.230	0.525 ± 0.020	2.672 ± 0.103
	3.468	0.461 ± 0.011	0.487 ± 0.019	2.449 ± 0.109	0.516 ± 0.021	2.565 ± 0.104
	6.935	0.695 ± 0.004	0.694 ± 0.015	2.469 ± 0.039	0.763 ± 0.015	2.648 ± 0.121
	10.403	1.762 ± 0.202	1.704 ± 0.088	2.475 ± 0.140	1.684 ± 0.062	2.546 ± 0.081
	17.339	4.557 ± 0.859	4.139 ± 0.173	16.066 ± 4.084	4.412 ± 0.234	15.337 ± 1.066
	34.677	9.385 ± 0.191	9.324 ± 0.212	14.148 ± 0.607	9.732 ± 0.481	17.010 ± 1.081
Tween 80	0.000 (water)	0.538 ± 0.038	0.502 ± 0.028	2.613 ± 0.140	0.575 ± 0.036	2.569 ± 0.164
	0.076	0.623 ± 0.034	0.520 ± 0.023	2.581 ± 0.077	0.646 ± 0.033	2.574 ± 0.102
	0.763	0.574 ± 0.039	0.531 ± 0.026	2.442 ± 0.055	0.573 ± 0.041	2.534 ± 0.148
	1.527	0.643 ± 0.054	0.588 ± 0.032	2.453 ± 0.062	0.636 ± 0.005	2.636 ± 0.009
	2.290	0.683 ± 0.052	0.683 ± 0.025	2.576 ± 0.073	0.714 ± 0.024	2.625 ± 0.060
	3.817	0.828 ± 0.021	0.783 ± 0.048	2.578 ± 0.045	0.902 ± 0.114	2.785 ± 0.279
	7.634	1.092 ± 0.083	1.035 ± 0.048	2.440 ± 0.023	1.154 ± 0.120	2.647 ± 0.124
	17.328	1.841 ± 0.149	2.097 ± 0.225	15.755 ± 0.253	2.177 ± 0.206	17.535 ± 4.597
	34.733	3.830 ± 0.095	3.871 ± 0.407	15.182 ± 0.410	3.789 ± 0.126	16.418 ± 1.112

Figure S1. Surface tension *versus* surfactant concentration. (**a**) SLS; (**b**) Tween 80.

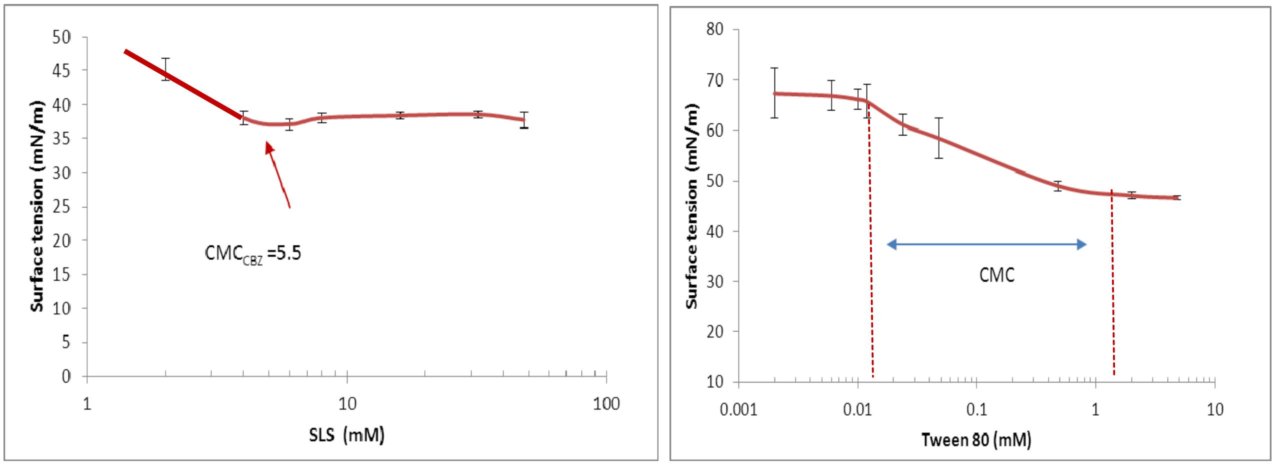

(**a**) SLS (**b**) Tween 80

Figure S2. DSC thermograms of solid residues at different NIC concentration solutions. (**a**) DSC thermograms in *Region I*; (**b**) DSC thermograms in *Region II*; (**c**) DSC thermograms in *Region III*; (**d**) DSC thermograms in region IV.

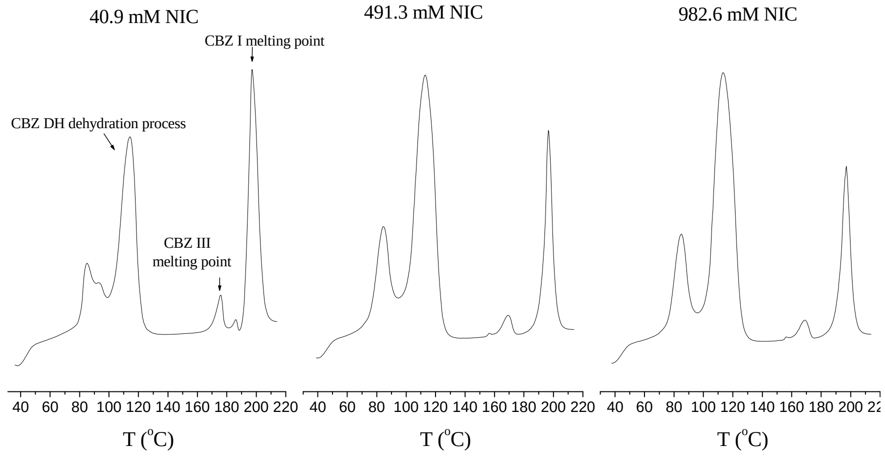

(**a**)

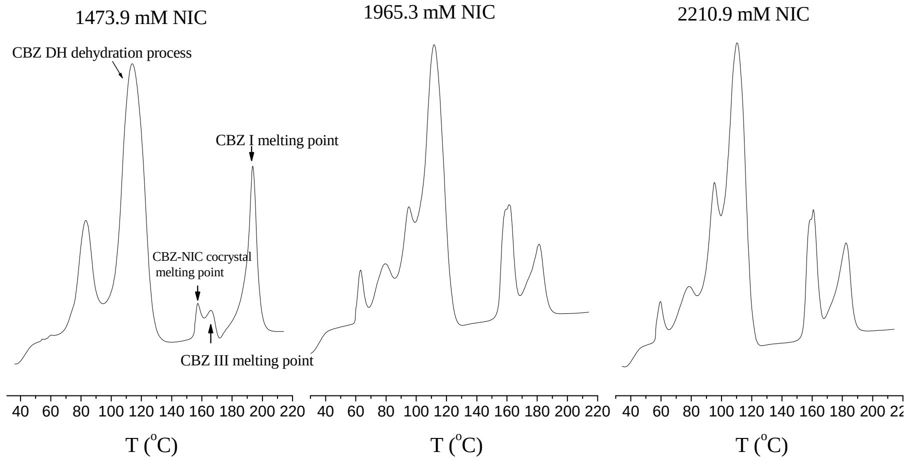

(**b**)

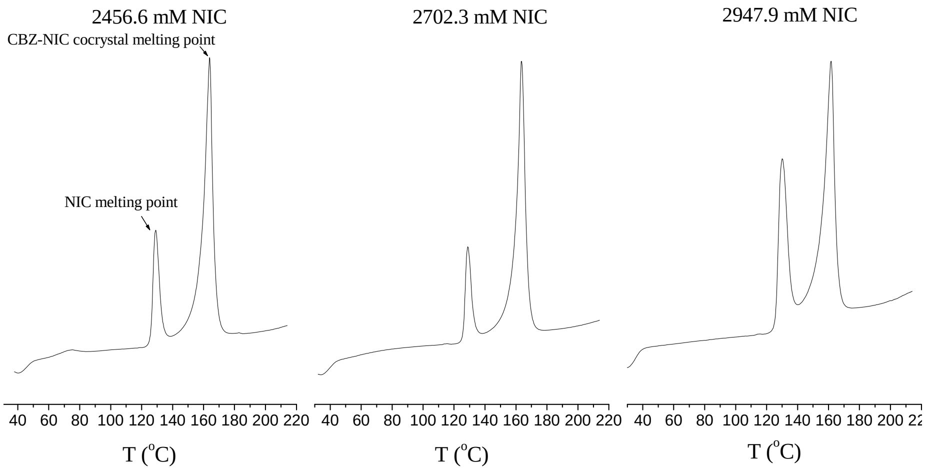

(**c**)

Figure S2. *Cont.*

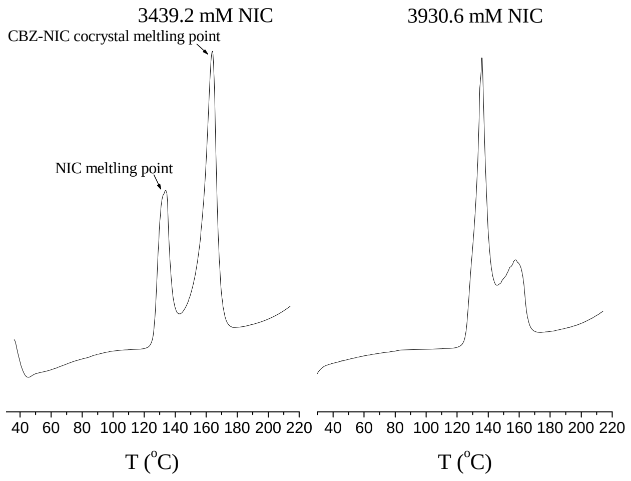

(**d**)

Figure S3. FTIR spectra of solid residues at different NIC concentration solutions.

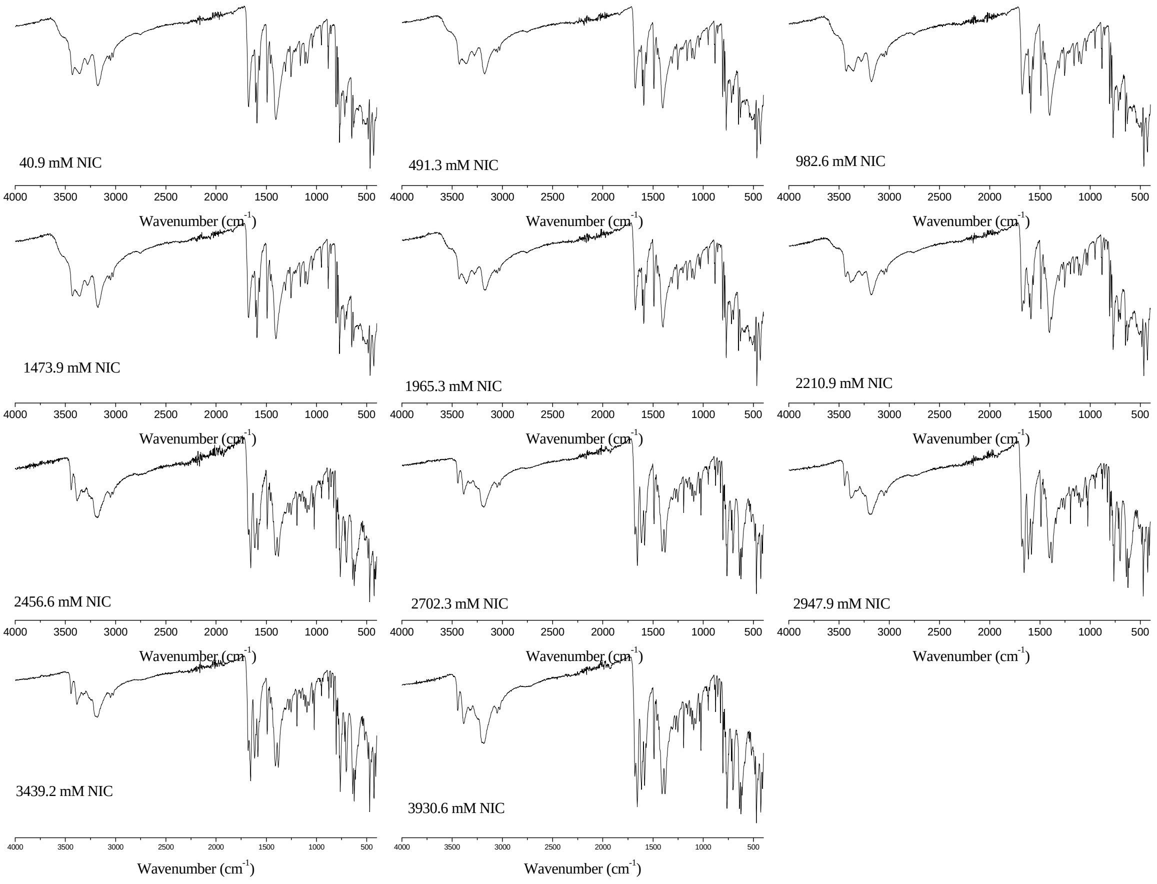

Figure S4. Raman spectra of solid residues at different NIC concentration solutions.

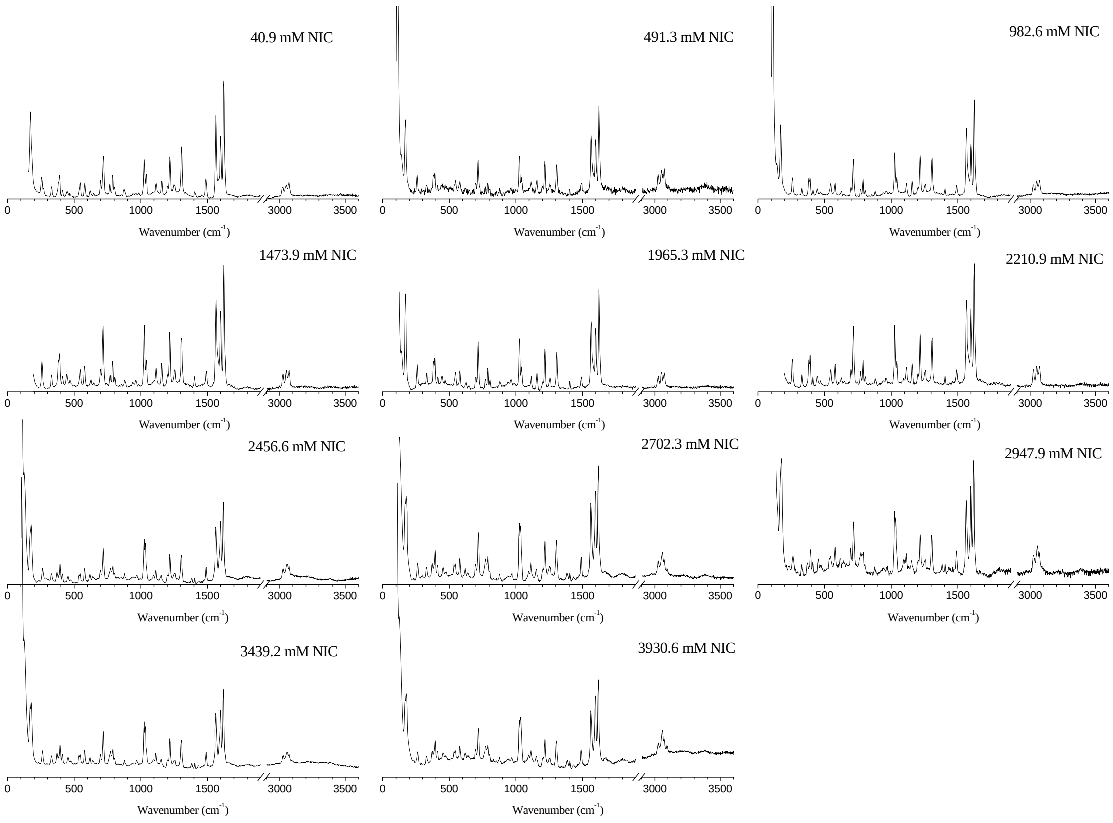

Figure S5. Raman spectra of the compacts before and after UV imaging dissolution at different dissolution media. (**a**) CBZ-NIC cocrystal; (**b**) CBZ III.

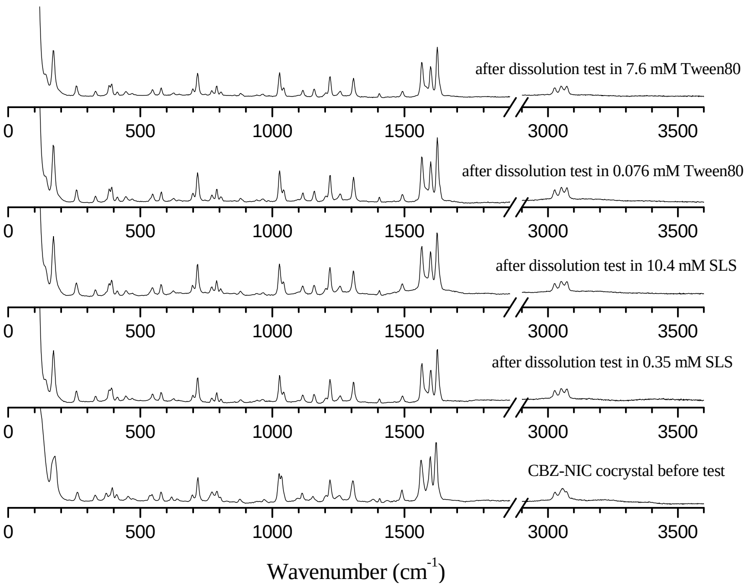

(**a**)

Figure S5. *Cont.*

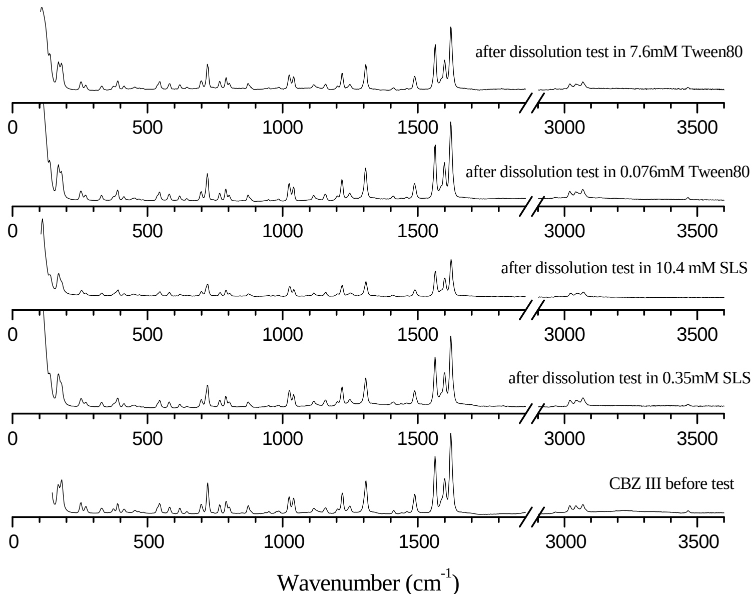

(**b**)

© 2013 by the authors; licensee MDPI, Basel, Switzerland. This article is an open access article distributed under the terms and conditions of the Creative Commons Attribution license (http://creativecommons.org/licenses/by/3.0/).
